# A novel mitosis-associated lncRNA, MA-linc1, is required for cell cycle progression and sensitizes cancer cells to Paclitaxel

**DOI:** 10.18632/oncotarget.4944

**Published:** 2015-08-11

**Authors:** Or Bida, Moriah Gidoni, Diana Ideses, Sol Efroni, Doron Ginsberg

**Affiliations:** ^1^ The Mina and Everard Goodman Faculty of Life Science, Bar Ilan University, Ramat Gan 52900, Israel

**Keywords:** lncRNA, cell cycle, Paclitaxel, E2F

## Abstract

Long noncoding RNAs (lncRNAs) are major regulators of many cellular processes including cell cycle progression and tumorigenesis. In this study, we identify a novel lncRNA, MA-linc1, and reveal its effects on cell cycle progression and cancer growth. Inhibition of MA-linc1 expression alters cell cycle distribution, leading to a decrease in the number of G1 cells and a concomitant increase in all other stages of the cell cycle, and in particular G2/M, suggesting its involvement in the regulation of M phase. Accordingly, knock down of MA-linc1 inhibits M phase exit upon release from a mitotic block. We further demonstrate that MA-linc1 predominantly functions in cis to repress expression of its neighboring gene, Purα, which is often deleted in human cancers and whose ectopic expression inhibits cell cycle progression. Knock down of Purα partially rescues the MA-linc1 dependent inhibition of M phase exit. In agreement with its suggested role in M phase, inhibition of MA-linc1 enhances apoptotic cell death induced by the antimitotic drug, Paclitaxel and this enhancement of apoptosis is rescued by Purα knockdown. Furthermore, high levels of MA-linc1 are associated with reduced survival in human breast and lung cancer patients.

Taken together, our data identify MA-linc1 as a novel lncRNA regulator of cell cycle and demonstrate its potential role in cancer progression and treatment.

## INTRODUCTION

Genome-wide transcriptome studies have revealed that mammalian genomes express thousands of long non-coding RNAs (lncRNAs), which are > 200 bases in length but lack significant open reading frames [1]. Thousands of lncRNAs are evolutionarily conserved [2, 3] and exhibit expression patterns that correlate with various cellular processes [2–6]. Furthermore, the expression of many lncRNAs is tissue-specific [1, 7] and many lncRNAs play a critical role in regulation of diverse cellular processes such as stem cell pluripotency, cell growth, development, differentiation and apoptosis [2–6, 8, 9]. It is now considered likely that this class of ncRNA represents a significant feature of normal cellular networks.

Recent studies have identified a number of mechanisms by which lncRNAs function [1, 10, 11]. Some well-characterized nuclear lncRNAs have been shown to modulate gene expression in cis by recruiting chromatin-modifying complexes and altering the chromatin structure of nearby target genes [12]. In contrast, other lncRNAs regulate gene expression in trans, mainly by directing the chromatin localization of associated proteins [1, 10, 11]. Their recognition of the target loci can involve recruitment by tethering proteins [12] or formation of RNA:DNA triplexes [13]. Some lncRNAs also exert indirect regulatory effects on gene expression by acting as decoys that sequester transcription factors [4, 14] or microRNAs [15–17].

Many lncRNAs are frequently aberrantly expressed in various human cancers, with potential roles in both oncogenic and tumor suppressive pathways [18–20]. Moreover, some lncRNAs induce epigenetic dysregulation of critical genes in cancer.

In particular, a number of lncRNAs were suggested to function in cell cycle progression via modulation of the expression of critical cell cycle regulators such as cyclins, CDKs and CDK inhibitors [21–24]. Other lncRNAs were shown to mediate p53-dependent cell cycle control [25, 26]. Interestingly, a recent study described a set of human lncRNAs that exhibit periodic expression during the cell cycle, and many of these are aberrantly expressed in cancer [4].

Many, although not all, lncRNAs are generated and processed through the same machinery as mRNA. Specifically, many lncRNAs are transcribed by RNA polymerase II [27] and their promoters are bound and regulated by transcription factors known to influence mRNA transcription, including cancer-related transcription factors such as p53 [28, 29], Myc [30, 31] and E2F [32–34].

Here, we demonstrate that a novel lncRNA, which we named MA-linc1 (Mitosis-Associated Long Intergenic Non-Coding RNA 1), is transcriptionally regulated by E2F1 and plays a role in cell cycle progression. Specifically, silencing of MA-linc1 in unsynchronized cells results in fewer cells in G1 and a concomitant increase in the number of cells in all other stages of the cell cycle, particularly in G2/M. Moreover, its silencing in M phase-arrested cells inhibits mitosis exit. The effect of MA-linc1 on cell cycle progression is mediated, at least in part, by repression of its neighboring gene, Purα, a cell cycle regulator whose expression induces cell cycle arrest. Importantly, high levels of MA-linc1 are correlated with decreased survival of breast and lung cancer patients and its silencing sensitizes cancer cells to the apoptotic effect of the M phase specific chemotherapeutic drug, Paclitaxel. This enhancement of Paclitaxel-induced apoptosis is also Purα-related.

## RESULTS

MA-linc1 (also termed ENST00000499203 (Ensembl) and Linc01024 (RefSeq)) was first identified as an E2F1-regulated lncRNA in an RNA Seq.-based screen we performed using the human osteosarcoma cell line U2OS and the human lung carcinoma cell line H1299, which express conditionally active E2F1 ([33]; Figure S1A). Next, we employed qPCR to analyze MA-linc1 levels in four cell lines: U2OS and H1299 cells, as well as the human embryonic lung fibroblasts, WI38, and another human osteosarcoma cell line, SAOS-2, each expressing the conditionally active E2F1. This analysis demonstrated that activation of the ectopic E2F1 resulted in a significant increase in MA-linc1 RNA levels in all four cell lines (Figure S1A, S1B). Activation of a mutated E2F1 that does not bind DNA did not affect MA-linc1 RNA levels, suggesting that E2F1 affects MA-linc1 RNA levels via a transcriptional mechanism (Figure S1A). Notably, activation of ectopic E2F3 also resulted in a significant increase in MA-linc1 RNA levels (Figure S1A). This suggests that an activity common to E2F1 and E2F3 is responsible for regulating MA-linc1 expression.

In support of direct regulation of MA-linc1 RNA levels, activation of ectopic E2F1 in the presence of cycloheximide did not mitigate MA-linc1 RNA induction (Figure S1C). Furthermore, the putative promoter of MA-linc1 contains two E2F consensus binding sites (Figure S2A), and indeed, E2F1 bound this putative promoter, as detected by ChIP analysis performed after activation of ectopic E2F1 (Figure S2B). Also, luciferase assay demonstrated that the DNA fragment upstream to MA-linc1 indeed functions as a promoter and its promoter activity is enhanced 3 fold by E2F1 (Figure S2C).

MA-linc1 is located on chromosome 5 (chr5:139482507-139487228) at a position corresponding to band 5q31.2 on the somatic map, it is transcribed from the minus strand and is composed of 3 exons.

**Figure 1:**
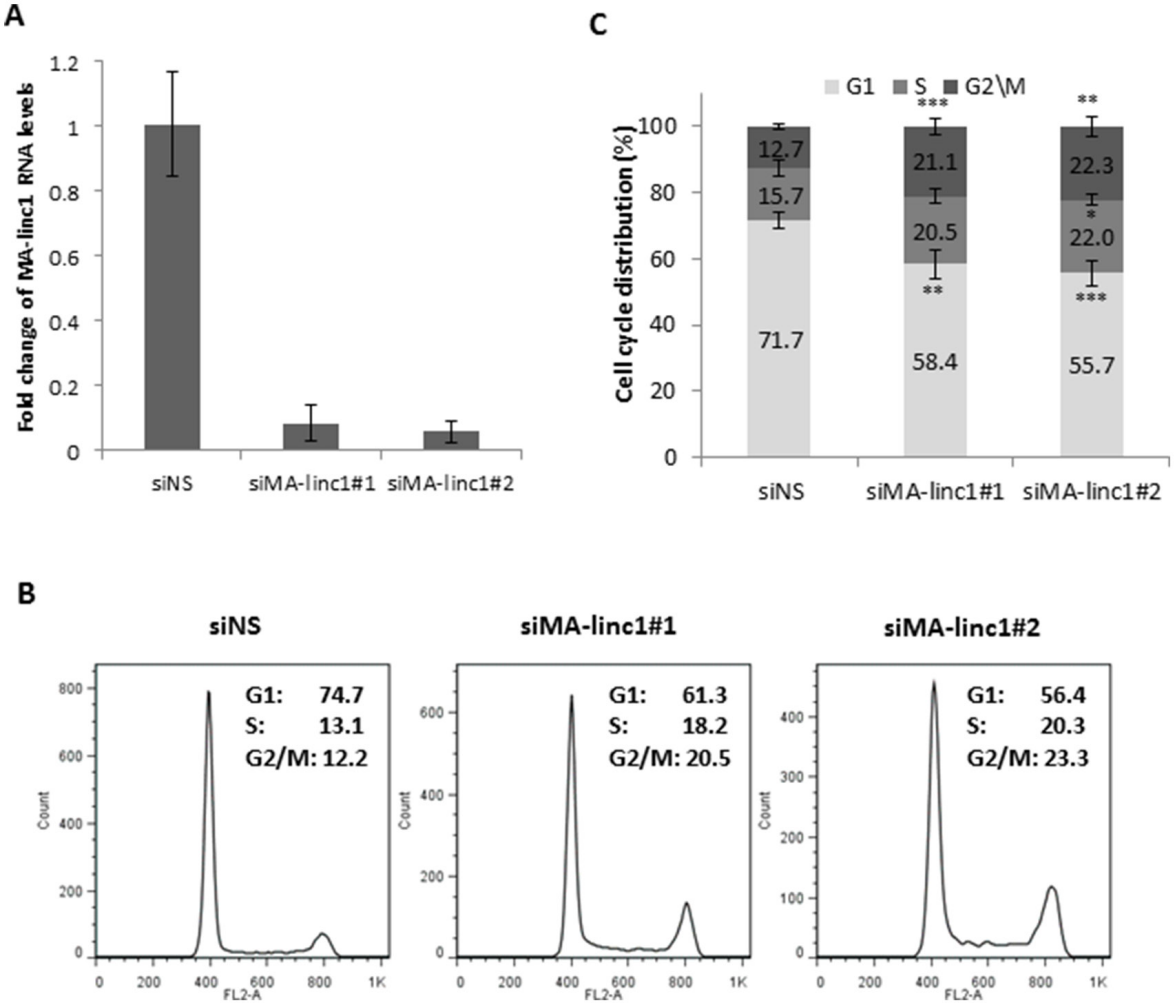
MA-linc1 affects cell cycle distribution U2OS cells were transfected with either a nonspecific siRNA (siNS) or an siRNA directed against MA-linc1 (siMA-linc1#1 or #2). Cells were harvested 48 post transfection. **A.** RNA was extracted, and MA-linc1 RNA levels were determined by real-time RT-PCR and normalized to GAPDH levels. Real-time RT-PCR experiments were performed in duplicates. One representative experiment is shown out of three independent experiments. **B.** and **C.** Cells described in (A) were analyzed by fluorescence-activated cell sorting (FACS) using Propidium-Iodide (PI) staining. (B) Cell cycle distribution histograms of a representative experiment. Percentages of cells in G1, S, and G2/M cell-cycle phases are depicted. (C) Average FACS analysis of three independent experiments. Percentages of cells in G1, S, and G2/M cell-cycle phases are depicted. (*P < 0.05, **P < 0.01, ***P < 0.005, two-tailed Students *t*-test).

**Figure 2:**
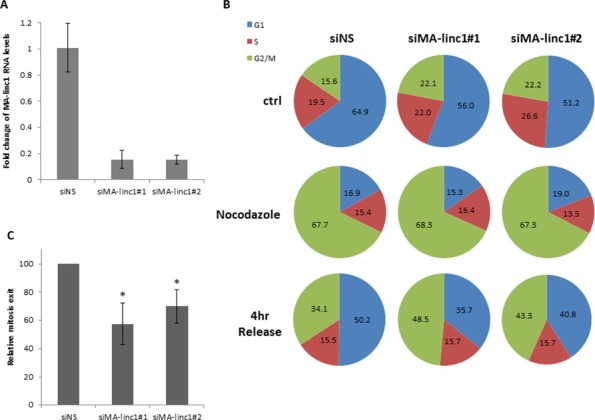
Silencing of MA-linc1 impairs M phase exit U2OS cells were transfected with either a nonspecific siRNA (siNS) or siRNA directed against MA-linc1 (siMA-linc1#1 or #2). Next, cells were left untreated or incubated with Nocodazole (60 ng/ml, 18 hr). The cells were then allowed to resume growth for 4 hours in fresh media. **A.** RNA was extracted, and MA-linc1 RNA levels were determined by real-time RT-PCR and normalized to GAPDH levels. Real-time RT-PCR experiments were performed in duplicates. **B+C.** Cells described in (A) were analyzed by FACS using Propidium-Iodide (PI) staining. (B) Cell cycle distribution is presented in pie charts. Percentages of cells in G1, S, and G2/M cell-cycle phases are depicted. The data represent an average of three independent experiments. (C) The average percentage of M phase exit of three independent experiments in a given sample, relative to the M phase exit in cells transfected with a nonspecific siRNA, which is depicted as 100 (*P < 0.05, two-tailed Student’s *t*-test).

Since many E2F-regulated genes encode proteins that affect cell cycle progression, we next tested whether MA-linc1 plays a role in this biological process. Indeed, reducing the endogenous MA-linc1 RNA levels in U2OS cells using two distinct siRNAs reproducibly resulted in cell cycle redistribution (Figure 1A, 1B). Specifically, upon knockdown of MA-linc1, we observed a significant decrease in the number of cells in G1 and a significant increase in the number of cells in all other stages of the cell cycle, on average a 35% increase in S phase and a 70% increase in G2/M (Figure 1B, 1C). We hypothesized that the G2/M enrichment detected upon knockdown of MA-linc1 may be due to an impairment of M phase exit. To directly determine whether MA-linc1 plays a role in M-phase exit, U2OS cells were arrested at M phase by Nocodazole and then induced to reenter the cell cycle by incubation in fresh media. Following Nocodazole treatment, the percentage of cells with a 4N DNA content rose to about 68%, and knockdown of MA-linc1, by two distinct siRNAs (Figure 2A), did not have a significant effect on this M-phase arrest (Figure 2B, middle). Importantly, the silencing of MA-linc1 impaired M phase exit; thus, after release from a Nocodazole-induced arrest, fewer MA-linc1 knocked-down cells exited M phase into G1, compared to cells transfected with a non-specific siRNA (Figure 2B). This impairment of M phase exit was reproducible and statistically significant (Figure 2C).

**Figure 3:**
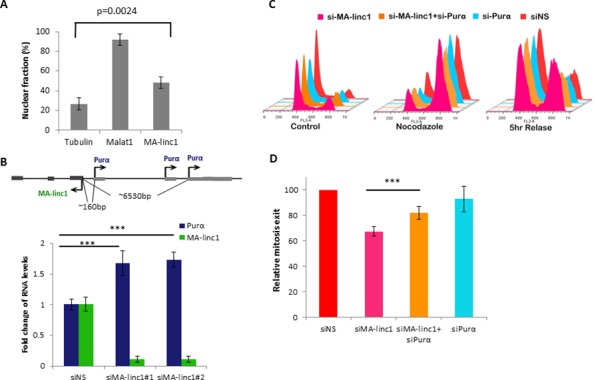
Purα knockdown partially rescues MA-linc1 -dependent M exit delay **A.** RNA was extracted from nuclear and cytoplasmic fractions of U2OS cells. The levels of MA-linc1 RNA in each fraction were determined by Real-time PCR. MALAT1 and Tubulin transcripts served as nuclear and cytoplasmic controls, respectively. The bar graph depicts the percentage of nuclear RNA out of whole cell RNA. Real-time PCR experiments were performed in duplicates. The bar graph presents an average of four independent experiments. **B.** Purα is upregulated upon MA-linc1 silencing. Upper panel - Schematic representation of human chromosome 5 at the MA-linc1 and Purα locus. Arrows indicate the transcription direction. Lower panel - U2OS cells were transfected with either a nonspecific siRNA (siNS) or a siRNA directed against MA-linc1 (siMA-linc1#1 or #2) and were harvested 48 post transfection. RNA was extracted and RNA levels of MA-linc1 and Purα were determined by real-time RT-PCR and normalized to GAPDH levels. Results shown are the average of three independent experiments. Real-time RT-PCR experiments were performed in duplicates (***P < 0.005). **C.** U2OS cells were transfected with either a nonspecific siRNA (siNS) or siRNA directed against MA-linc1 (siMA-linc1), Purα (siPurα) or both (siMA-linc1+siPurα). Next, cells were left untreated or incubated with Nocodazole (60 ng/ml) for 18 hours. Then cells were allowed to resume growth for 5 hours in fresh media. Cells were then analyzed by FACS using Propidium-Iodide (PI) staining. **D.** The average percentage of M phase exit of five independent experiments in a given sample, relative to the M phase exit in cells transfected with a nonspecific siRNA, which is depicted as 100 (***P < 0.005, two-tailed Students *t*-test).

In an attempt to elucidate the molecular mode of action of MA-linc1, we first studied its sub-cellular localization. Cell fractionation experiments indicated that MA-linc1 is present both in the nucleus and the cytoplasm of cells (Figure 3A). In recent years, a number of well-characterized nuclear lncRNAs have been shown to function via modulation of gene expression in cis [35]. Therefore, we focused on the nearest neighboring gene of MA-linc1, Purα, which is located upstream to MA-linc1 in a head-to-head orientation. Purα encodes a single-stranded DNA- and RNA-binding protein, which binds a purine rich element that is often present at origins of replication and in gene flanking regions of eukaryotes [36]. MA-linc1 and Purα are both regulated by E2F1 (Figure S2 and [37]). qPCR analysis demonstrated that silencing of MA-linc1 by two distinct siRNAs results in a moderate increase in Purα mRNA levels (Figure 3B), suggesting that MA-linc1 represses the expression of its neighboring gene, Purα. Of note, this not only suggests that MA-linc1 affects gene expression in cis, but also offers insight to the mechanism underlying the effect of MA-linc1 on cell cycle progression, as Purα was shown to arrest cell cycle progression [38, 39].

To test more directly whether MA-linc1 affects cell cycle progression via modulation of Purα levels, we examined the effects of silencing both genes simultaneously. As can be seen in Figure 3B, the simultaneous knock down of MA-linc1 and Purα also did not have any detectable effect on Nocodazole-induced cell cycle arrest. Importantly, while silencing of Purα alone did not affect re-entry to the cell cycle, its simultaneous silencing (together with MA-linc1) partially rescued the impaired re-entry to the cell cycle observed upon MA-linc1 silencing (Figure 3C). This partial rescue was reproducible and statistically significant (Figure 3D). These data indicate that MA-linc1 affects cell cycle progression, at least in part, via regulating the levels of its neighbor, Purα.

**Figure 4:**
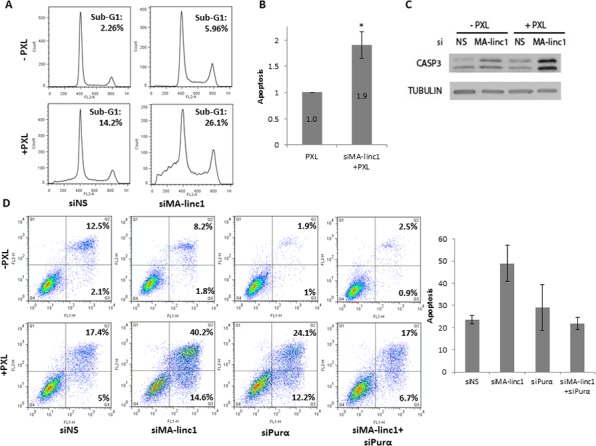
Silencing of MA-linc1 enhances Paclitaxel-induced apoptosis U2OS cells were transfected with either a nonspecific siRNA (siNS) or siRNA directed against MA-linc1 (siMA-linc1). Then, cells were treated with 12 nM Paclitaxel (PXL) for 42 hr or left untreated. Cells were harvested 48 post transfection. **A.** Cells were analyzed by FACS using Propidium-Iodide (PI) staining. The percentage of cells with sub-G1 DNA content is indicated. **B.** The average apoptotic fractions of three independent FACS experiments, relative to the apoptosis in Paclitaxel treated cells transfected with a nonspecific siRNA, which is depicted as 1 (*P < 0.05, two-tailed Student’s *t*-test). **C.** Protein extracts from cells described in A were subjected to western blot analysis using antibodies against cleaved caspase-3 and tubulin. **D.** U2OS cells were transfected for 72 hours with either a nonspecific siRNA (siNS) an siRNA directed against MA-linc1 (siMA-linc1), Purα (siPurα) or both (siMA-linc1+siPurα). Cells were treated with 20 nM Paclitaxel (PXL) for the last 66 hr prior to harvesting or left untreated. Next, cells were stained with Annexin-V FITC and PI and analyzed for apoptosis by flow cytometric analysis. Percentage of early and late apoptotic cells are indicated. The average of two independent experiments is presented in bar graph.

A number of anti-cancer drugs induce cell death via inhibition of either the polymerization and depolymerization of microtubules during M phase [40]. Since silencing of MA-linc1 increases the percentage of cells in M phase, we speculated that it may enhance the cellular response to such drugs. The knockdown of MA-linc1 alone did not cause significant cell death, and less than 6% of the cells exhibited a Sub-G1 DNA content (Figure 4A, top right panel). Administration of sub-lethal doses of Paclitaxel, an anti-cancer drug that inhibits the depolymerization of microtubules, induced some apoptosis; 14% of the cells underwent apoptosis after 42 hr (Figure 4A, bottom left panel). Combining these sub-lethal doses of Paclitaxel with MA-linc1 silencing resulted in substantially higher levels of apoptosis, of 26% (Figure 4A bottom right panel). On average, the silencing of MA-linc1 led to a 90% increase in apoptosis (Figure 4B). Similar results were obtained upon knock down by another siRNA directed against MA-linc1 (data not shown). In line with the data obtained by FACS, increased cleavage of caspase 3 was detected after the combined treatment of MA-linc1 silencing plus administration of Paclitaxel (Figure 4C). Moreover, similar data were obtained when we employed an Annexin V/PI analysis. Specifically, 22% of the cells underwent apoptosis 66 hr after administration of Paclitaxel and combining this dose of Paclitaxel with MA-linc1 silencing resulted in apoptosis of 54% of the cells (Figure 4D). Importantly, while silencing of Purα alone did not inhibit Paclitaxel-induced apoptosis, its simultaneous silencing (together with MA-linc1) impaired the enhancement of Paclitaxel-induced apoptosis observed upon MA-linc1 silencing (Figure 4D). These data further support the notion that MA-linc1 exerts its biological function, at least in part, via an effect on its neighbor, Purα.

**Figure 5:**
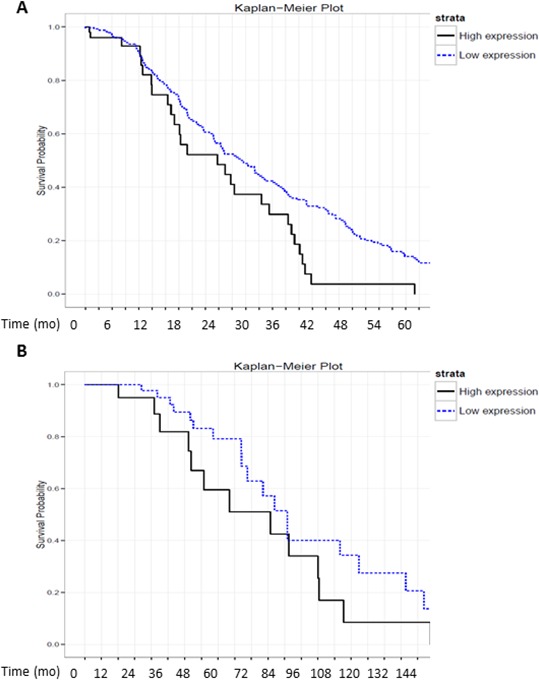
High levels of MA-linc1 are associated with poor prognosis in breast cancer and lung cancer patients **A+B.** RNA-seq. data derived from 355 lung cancer and 90 breast cancer samples were each stratified into two groups according to the RNA levels of MA-linc1 (high vs. low). (A) 60 lung cancer patients with high expression (median= 184) and 295 with low expression levels (median = 61) *P* < 0.02. (B) 31 breast cancer patients with high expression (median= 196) and 59 with low levels (median = 96) *P* < 0.05. The survival data of the two subgroups is presented in Kaplan–Meier survival curves.

Finally, to evaluate the physiological relevance of MA-linc1 in human tumors, we tested whether its levels correlate with patient survival in a cohort of 355 Lung cancer patients and in a cohort of 90 Breast cancer patients. Remarkably, upon stratification of the lung cancer cohort into two subgroups: one with high levels of MA-linc1 and the other with low levels of MA-linc1, it became apparent that the group with low levels of MA-linc1 exhibited significantly increased survival (Figure 5A). Similar results were obtained when analyzing the breast cancer cohort of 90 patients (Figure 5B). These data are consistent with the findings that low MA-linc1 levels lead to inhibition of cell cycle progression (shown in Figures 1, 2).

## DISCUSSION

Long non coding RNAs are emerging as important regulators of many biological processes including cell cycle progression and tumorigenesis [18, 41]. We report here the identification of a novel lncRNA, MA-linc1, that affects cell cycle progression. In agreement with a possible role in M phase exit, the silencing of MA-linc1 sensitizes cancer cells to Paclitaxel, a chemotherapeutic drug that activates the mitotic checkpoint leading to apoptotic cell death [40]. Furthermore, we show here that high levels of MA-linc1 are associated with poor prognosis in breast and lung cancer.

### The E2F1-regulated MA-linc1 is a modulator of cell cycle progression

E2Fs are transcription factors best known for their involvement in the timely regulation of protein-coding genes required for cell cycle progression [42]. Though E2F1 is particularly known as a regulator of the G1/S transition, a number of pivotal mitotic regulators are transcriptionally activated by E2Fs [43–45]. Recent studies indicate that E2Fs also regulates the expression of non-coding RNAs, including microRNAs and lncRNAs that control cell cycle progression [34, 46–48]. Thus far, three lncRNAs were shown to exhibit E2F-regulated expression. These are H19, a lncRNA encoded by an imprinted gene that exhibits remarkably elevated levels in a large number of human cancers [32]; ANRIL, which is located at the tumor suppressor locus encoding p16^INK4A^ and p15^INK4B^ and represses the expression of these two tumor suppressors [21, 34, 49]; and ERIC, which was shown to regulate apoptosis that is induced by either E2F1 or DNA damage [33]. MA-linc1 now joins this short list of E2F-regulated lncRNAs, and our data indicate that like ANRIL it plays a role in cell cycle progression. Of note, our results do not exclude the possibility that MA-linc1 also affects the G1/S transition, as its silencing in unsynchronized cells leads to a decrease in the number of cells in G1 and a concomitant increase in number of cells in S phase. Nevertheless, we detected a prominent effect of its silencing on M phase. Specifically, upon silencing of MA-linc1, fewer cells were released from mitotic checkpoint arrest and proceed through M phase into a new cell cycle.

### MA-linc1 affects M-phase, at least in part, by regulating the expression of its neighbor, Purα

Many lncRNAs act near their site of synthesis to regulate the expression of genes in *cis*, often by binding to chromatin modifiers that affect a neighboring gene(s) [1, 11, 12]. Our data indicate that MA-linc1 also affects the expression of its neighboring gene, Purα which is located upstream to MA-linc1. The molecular mechanism(s) underlying this regulation remain to be determined.

Purα is transcribed, in a head to head orientation to MA-linc1, from three distinct promoters; its most upstream TSS is about 160 bases upstream to MA-linc1 while its most downstream promoter is 6Kb upstream to MA-linc1 [50]. The expression of MA-linc1 may represent a divergent transcription to that of Purα from its most upstream promoter, a situation which is observed for many lncRNAs [51].

Purα is a regulator of cell proliferation; its ectopic expression causes cell cycle arrest at either the G1/S or G2/M transition points [52] and suppresses the growth of several transformed and tumor cells including glioblastomas [53]. Thus, its regulation by MA-linc1 provides a molecular mechanism underlying the effect of MA-linc1 silencing on M phase exit. Indeed, co-silencing of MA-linc1 and Purα partially rescues the M phase exit defect exhibited by MA-linc1 knocked-down cells.

This Purα-dependent rescue is partial, indicating that MA-linc1 most probably has additional targets. In agreement with this notion, our analysis of the transcriptome after silencing of MA-linc1 show that in addition to Purα the expression of a number of proliferation-related genes is altered. The detailed effects of MA-linc1 on expression of these genes expression and their relevance to the effects of MA-linc1 on cell cycle progression awaits further studies.

### MA-linc1 affects cancer patient survival and the response of cancer cells to Paclitaxel

In agreement with a possible role for MA-linc1 in M phase, we show here that while silencing of MA-linc1 causes little to no apoptosis, it sensitizes cancer cells to the anti-cancer chemotherapeutic drug Paclitaxel, which inhibits the depolymerization of microtubules. Importantly, our data strongly suggest that the effect of MA-linc1 silencing on Paclitaxel-induced apoptosis is also mediated by its effects on its neighboring gene Purα. Taken together with the fact that Purα is often deleted in some human cancers [38], this data suggests that MA-linc1 may affect the initiation and progression of cancer by regulating Purα levels.

Paclitaxel is commonly used to treat various cancers including Breast, Ovarian, and Lung cancer [40]. Potentially, a clinically relevant corollary of our findings is that tumors expressing low levels of MA-linc1 may be more sensitive to treatment with Paclitaxel, and perhaps to additional anti-cancer drugs that affect microtubule dynamics.

More generally, analysis of breast and lung cancer patients suggests that this lncRNA also impacts cancer biology, in particular, survival irrespective of chemotherapeutic treatment, as in both cohorts patients with low levels of MA-linc1 lived longer.

In summary, our data suggest that silencing of MA-linc1 may benefit cancer patients and specifically, may improve their response to chemotherapeutic drugs, such as Paclitaxel, that attack cells at the M phase.

## MATERIALS AND METHODS

### Cell culture

U2OS and SAOS-2 osteosarcoma cells were grown in Dulbecco’s modified Eagle’s medium supplemented with 5% fetal bovine serum (FBS). Early passage WI38 human embryonic lung fibroblasts were grown in minimal essential medium supplemented with 10% fetal bovine serum, 1 mM L-glutamine, 1 mM sodium pyruvate and non-essential amino acids. H1299 human lung adenocarcinoma cells were grown in RPMI 1640 medium supplemented with 5% fetal bovine serum. Cells were maintained at 37°C in a humidified atmosphere containing 8% CO_2_. To induce activation of ER-E2F1 or ER-E2F3, cells were treated with 100 nM 4-hydroxytamoxifen (OHT, Sigma) for the times indicated. Where indicated, cycloheximide (CHX, Sigma) was administered for 8 hr at 10 μg/ml. Nocodazole (Sigma) was used at 60 ng/ml for 18 hr.

### Quantitative PCR (Real-Time RT- PCR)

Total RNA was extracted from the cells using the Tri Reagent method. To obtain cDNA, RT was performed using cDNA synthesis kit (Quanta). Real-time quantitative PCR (qPCR) was performed using PerfeCTa SYBR Green fastMix (Quanta) with the following primer pairs:

GAPDH: 5′-CATGTTCCAATATGATTCCACC-3′ and 5′-GATGGGATTTCCATTGATGAC-3′

MA-linc1:5′-TCATCCCAGTTAAAATGGCTTT-3′ and 5′-TTTCGGAGGCACTTCCATAC-3′

Purα: 5′-GACGACTACGGAGTGGAGGA-3′ and 5′-TCGCTCACTCGCATAAACAC-3′

Real-Time PCR was performed and analyzed in The StepOnePlus™ Real-Time PCR System (Applied Biosystems). Results are presented as mean and SD for duplicate runs.

### Western blotting

Cells were lysed in lysis buffer [50 mL Tris (pH 7.5), 150 mL NaCl, 1 mL EDTA, 1% NP40] in the presence of Aprotinin, Leupeptin, Phenylmethanesulfonyl fluoride (PMSF) (Sigma) and phosphatase inhibitor cocktails II and III (Sigma). Equal amounts of protein, as determined by the Bradford assay, were resolved by electrophoresis through a 12.5% polyacrylamide SDS gel and then transferred to a PVDF membrane (Millipore). The membrane was incubated with the following primary antibodies: anti-cleaved caspase-3 (9664, Cell Signaling) and anti-tubulin (T9026, Sigma). Binding of the primary antibody was detected using an enhanced chemiluminescence kit (ECL Amersham).

### Transfection

Transfections of siRNA were performed using the Interferin transfection reagent (PolyPlus-transfection) according to the manufacturer’s instructions. The siRNAs against MA-linc1 (siMA-linc1#1: AAGAGTGGATCTATCTGAACTGGAT, siMA-linc1#2: AAACTGTATGGAAGTGCCTCCGAAA), Purα (siPurα: GGCTCCAACAAGTACGGCGTGTTTA), and a control sequence (siRNA universal negative control #1), were synthesized by Sigma-Aldrich. Experiments were performed 48 hours following siRNAs transfection.

### Chromatin immunoprecipitation

DNA–protein complexes were immunoprecipitated from U2OS cells using the ChIP (chromatin immunoprecipitation) assay kit (Upstate Biotechnology) according to the manufacturer’s protocol with the following antibodies: anti-E2F1 (sc-251; Santa Cruz Biotechnology) and anti-IgG (111-035-144, Jackson Immunoresearch). Anti-IgG served as a control for nonspecific DNA binding. The precipitated DNA was subjected to RT- PCR analysis using specific primers corresponding to the estimated human MA-linc1 promoter (5′-GGGCTGAGGAGGAAGGAG-3′ and 5′- GACGTCGCCTGGAGTCAC -3′) as well as primers for β-actin, which served as a negative control (5′-ACGCCAAAACTCTCCCTCCTCCTC-3′ and 5′-CATAAAAGGCAACTTTCGGAACGGC-3′).

### Fluorescence-activated cell sorting (FACS) analysis

For PI staining, cells were trypsinized and then fixed by incubating in 70% ethanol at 4°C overnight. After fixation, cells were centrifuged for 4 min at 1500 rpm, and the pellet resuspended and incubated for 40 min at 4°C in 1 ml of PBS. Then, the cells were centrifuged again and resuspended in PBS containing 5 mg/ml propidium iodide and 50 μg/mL RNase A. After incubation for 20 min at room temperature, fluorescence intensity was analyzed using a Becton Dickinson flow cytometer.

For Annexin V/PI staining, cells were collected, stained with annexin-V/PI according to the manufacturer’s instructions (MBL, MEBCYTO Apoptosis Kit) and analyzed by flow-cytometry (FACS calibur, BD).

### Extraction of nuclear and cytoplasmic RNA

RNA was extracted from nucleus and cytoplasm according to the Invitrogen nuclear extraction protocol. Cells were washed twice with PBS and resuspended in 500 μl of Hypotonic Buffer followed by 15 minutes incubation on ice. Then, 10% NP40 detergent was added and the homogenate was centrifuged for 10 minutes at 3,000 rpm at 4°C. The RNA from the pellet, containing the nuclear fraction, was extracted by the Tri Reagent method. The RNA from the supernatant, containing the cytoplasm fraction, was extracted by the Phenol-Chloroform method.

RNA levels of the nuclear and the cytoplasmic fractions were monitored by RT Real Time PCR and were normalized to levels of external DNA.

### Reporter gene assay

Dual-Luciferase assay was performed in U2OS cells transfected with PolyJet *In Vitro* DNA Transfection Reagent. Cells were harvested 48 hours post-transfection and assayed for Dual-Luciferase activities as specified by the manufacturer (Promega). The firefly luciferase activity of each sample was normalized to the corresponding Renilla luciferase activity. Each transfection was performed in triplicate.

### Cloning

Human genomic DNA was subjected to PCR analysis using specific primers corresponding to the MA-linc1 promoter (5′-CTTAGGCTCTGCGGGCTGAG GAGGAAGGAG-3′ and 5′-CCTGGCCCGCGGAATGTT GACG-3′). Sequence-verified MA-linc1 promoter was cloned into firefly luciferase reporter.

### Clinical data and survival probability analysis

For the survival analysis, two large RNA Next Generation Sequencing (NGS) datasets of Breast invasive carcinoma (BRCA) and Lung Adeno Carcinoma (LUAD) tumor samples were downloaded from The Cancer Genome Atlas (TCGA).

These datasets included 90 unaligned and 355 pre-aligned samples of BRCA and LUAD respectively. RSEM (http://www.biomedcentral.com/1471-2105/12/323), an RNA-seq aligner, was implemented by TCGA for LUAD dataset alignment. Therefore, for BRCA samples we used the same aligner with standard parameters, hg19 reference genome annotation and a custom GTF file, which included annotation of known and predicted lncRNAs in addition to all known genes. Next, transcript count tables for each dataset were generated. In the transcript count table, the rows (i) represent transcripts and the columns (j) samples; each cell (i,j) represents the raw abundance of reads aligned to a transcript (i) in the aligned sample (j). These tables, which are required for further normalization and processing, were constructed differently for LUAD and BRCA datasets. For the BRCA dataset, we extracted the transcript abundance from the RSEM output, while for the LUAD dataset, a transcript count table was created using FeatureCounts [54] with a custom GTF file that was used for BRCA alignment.

Next, transcript counts tables of both datasets were normalized by using DESeq [55], a Bioconductor package [56]. Further, each dataset was divided into two groups according to MA-linc1 expression, “high” and “low”. The clinical data on the survival of the patients was also downloaded from TCGA, and was used for Kaplan–Meier survival analysis.

## SUPPLEMENTARY MATERIAL FIGURES


