# Supplementary material for: A novel mitosis-associated lncRNA, MA-linc1, is required for cell cycle progression and sensitizes cancer cells to Paclitaxel
Source: Oncotarget. 2015 Aug 11;6(29):27880–90. doi: 10.18632/oncotarget.4944 (PMC4695032; doi:10.18632/oncotarget.4944)
Supplement: Supplementary file 1 [file oncotarget-06-27880-s001.pdf]

SUPPLEMENTARY FIGURES

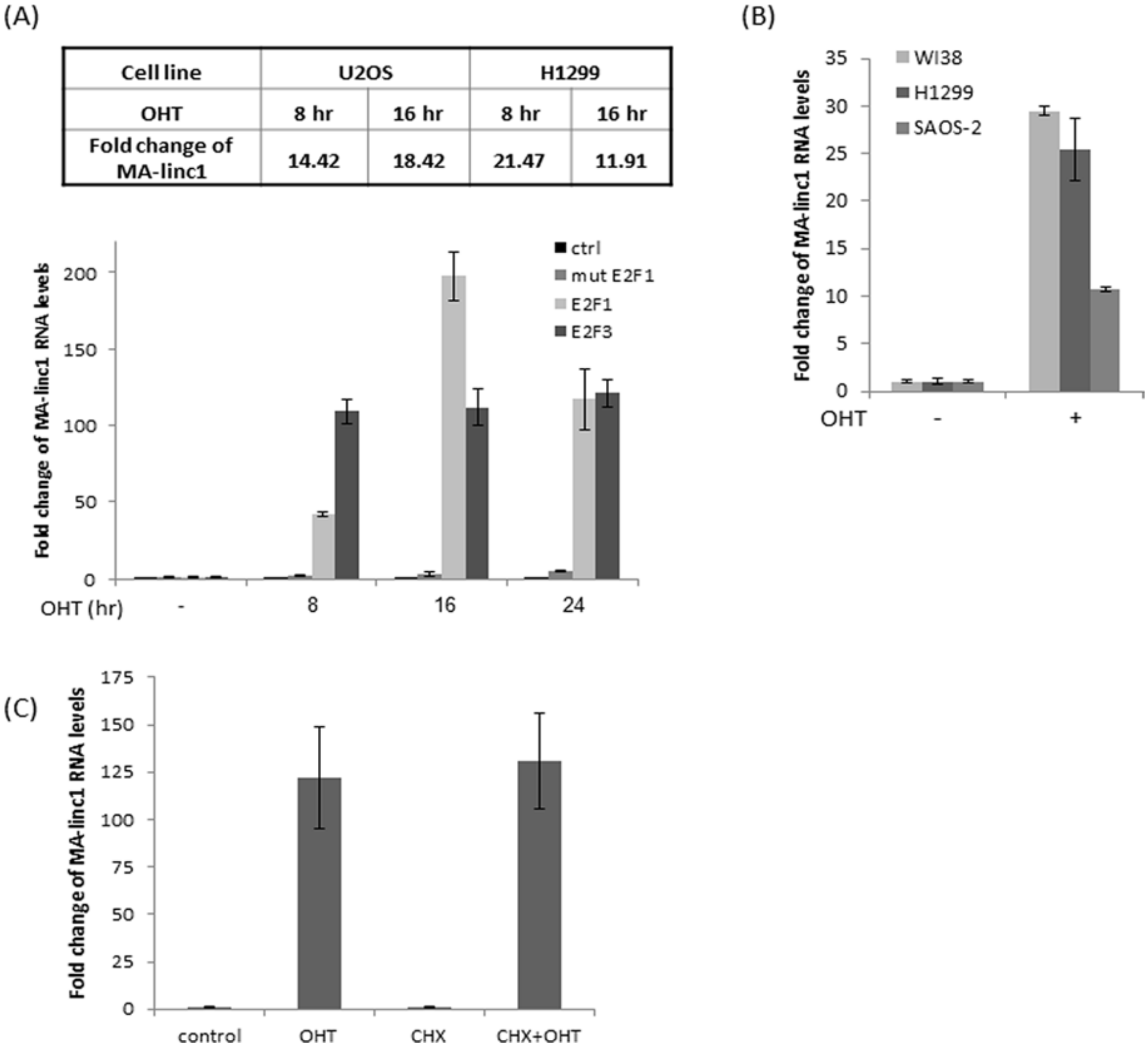

Cell line	U2OS		H1299	
OHT	8 hr	16 hr	8 hr	16 hr
Fold change of MA-linc1	14.42	18.42	21.47	11.91

Supplementary Figure S1: Ectopic E2F1 expression directly upregulates MA-linc1 RNA levels. A. Upper panel - U2OS and H1299 cells expressing conditionally active E2F1 were induced to activate E2F1 by incubation with 4-hydroxytamoxifen (OHT; 100 nmol/L) for the times indicated. RNA was extracted and RNA deep-sequencing analysis was employed. Lower panel - U2OS cells, uninfected (ctrl) or infected with a vector expressing either ER-E2F3 (E2F3), wild type ER-E2F1 (E2F1) or mutant ER-E2F1 (mut E2F1), were incubated with OHT for the times indicated, or left untreated. RNA was extracted and MA-linc1 RNA levels determined by Real-time RT-PCR and normalized to GAPDH. All real-time RT-PCR experiments were carried out in duplicates. **B.** SAOS, H1299 and WI38 cells expressing ER-E2F1, were treated with OHT for 8 hours (+) or left untreated (−). RNA was extracted and MA-linc1 RNA levels determined by Real-time RT-PCR and normalized to GAPDH levels. **C.** Induction of MA-linc1 does not require protein synthesis. U2OS cells expressing ER-E2F1 were left untreated or incubated with OHT (100 nM), cycloheximide (CHX; 10 μg/mL) or both (OHT+CHX) for 8 hours. RNA was extracted and MA-linc1 RNA levels determined by real-time RT-PCR and normalized to GAPDH.

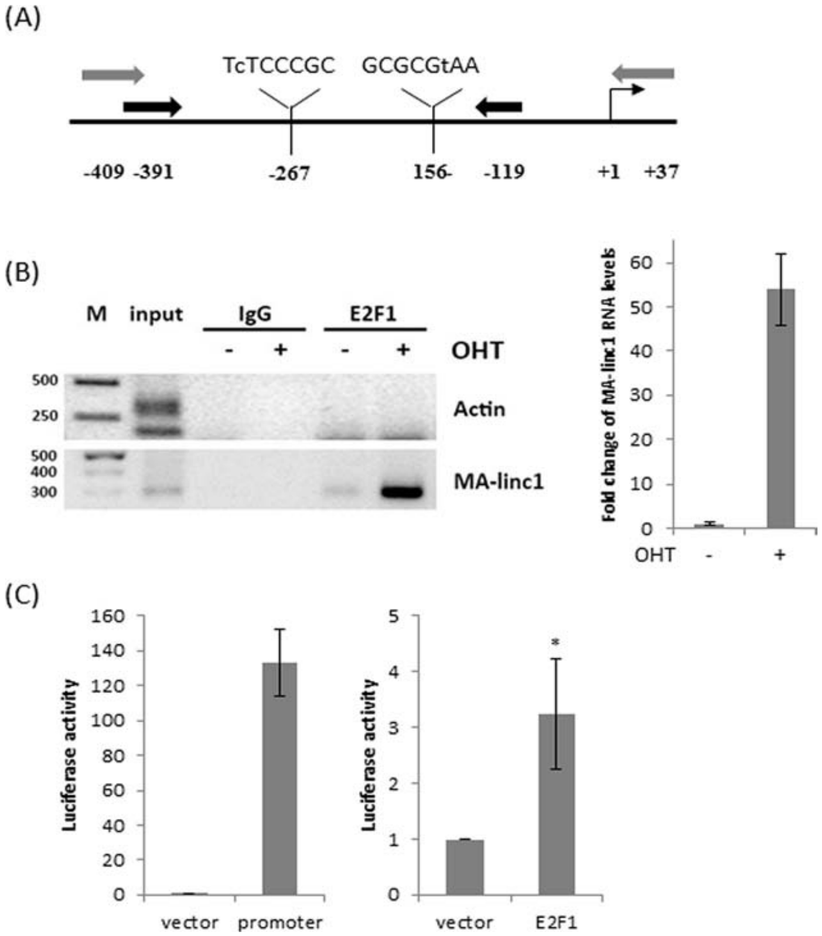

Supplementary Figure S2: E2F1 binds and activates MA-linc1 promoter. A. Schematic representation of the human MA-linc1 promoter. The E2F-binding sites are represented as 8-mer nucleotide sequences. Lower case letters represent mismatches from the consensus. The transcription start site (+1) is indicated by an arrow (⟶). The DNA fragments amplified by PCR for the ChIP and luciferase are represented by arrows (➡ −391/−119 or ➡ −409/+37, respectively). **B.** U2OS cells expressing conditionally active E2F1 were incubated with OHT (100 nM) for 6 hours or left untreated. Left panel- ChIP analysis was conducted. Cross-linked chromatin was precipitated using either antibodies directed against E2F1 or IgG. Then, MA-linc1 and actin promoter fragments were amplified by PCR. Input DNA represents 0.5% of total chromatin. Right panel- RNA was extracted and MA-linc1 RNA levels determined by Real-time RT-PCR and normalized to GAPDH. **C.** Left panel- U2OS cells were transfected with firefly luciferase reporter plasmid containing the MA-linc1 promoter (promoter) or not (vector). Right panel- cells were co-transfected with E2F1 expressing vector (E2F1) or a control vector (Vector) as well as a firefly luciferase reporter gene downstream to the MA-linc1 promoter. Cell extracts were assayed for Dual-Luciferase activity. To normalize for variations in transfection efficiency, cells were co-transfected with a Renilla luciferase control plasmid. An average of four independent experiments is presented (*$P < 0.05$)
